# Long non‐coding RNA SNHG16 regulates human aortic smooth muscle cell proliferation and migration via sponging miR‐205 and modulating Smad2

**DOI:** 10.1111/jcmm.14576

**Published:** 2019-08-23

**Authors:** Yongqing Lin, Guoping Tian, Haifeng Zhang, Woliang Yuan, Yong Xie, Ying Yang, Jingfeng Wang, Ying Liang

**Affiliations:** ^1^ Department of Cardiology Sun Yat‐sen Memorial Hospital, Sun Yat‐sen University Guangzhou China; ^2^ Department of Cardiovascular Medicine The Second Affiliated Hospital of University of South China Hengyang China; ^3^ Department of Endocrinology Sun Yat‐sen Memorial Hospital, Sun Yat‐sen University Guangzhou China

**Keywords:** atherosclerosis, migration, miR‐205, proliferation, Smad2, SNHG16

## Abstract

The present study investigated the role of long non‐coding RNA (lncRNA) small nucleolar RNA host gene 16 (SNHG16) in the human aortic smooth muscle cell (HASMC) proliferation and migration and explored the potential link between SNHG16 and atherosclerosis. Our results showed that platelet‐derived growth factor (PDGF)‐bb treatment promoted cell proliferation and migration with concurrent up‐regulation of SNHG16 in HASMCs. Small nucleolar RNA host gene 16 overexpression promoted HASMC proliferation and migration, while SNHG16 knockdown suppressed cell proliferation and migration in PDGF‐bb‐stimulated HASMCs. The bioinformatic analyses showed that SNHG16 possessed the complementary binding sequence with miR‐205, where the interaction was confirmed by luciferase reporter assay and RNA pull‐down assay in HASMCs, and SNHG16 inversely regulated miR‐205 expression. MiR‐205 overexpression attenuated the enhanced effects of PDGF‐bb treatment on HASMC proliferation and migration. Moreover, Smad2 was targeted and inversely regulated by miR‐205, while being positively regulated by SNHG16 in HASMCs. Smad2 knockdown attenuated PDGF‐bb‐mediated actions on HASMC proliferation and migration. Both miR‐205 overexpression and Smad2 knockdown partially reversed the effects of SNHG16 overexpression on HASMC proliferation and migration. Moreover, SNHG16 and Smad2 mRNA were up‐regulated, while miR‐205 was down‐regulated in the plasma from patients with atherosclerosis. Small nucleolar RNA host gene 16 expression was inversely correlated with miR‐205 expression and positively correlated with Smad2 expression in the plasma from atherosclerotic patients. In conclusion, our data showed the up‐regulation of SNHG16 in pathogenic‐stimulated HASMCs and clinical samples from atherosclerotic patients. Small nucleolar RNA host gene 16 regulated HASMC proliferation and migration possibly via regulating Smad2 expression by acting as a competing endogenous RNA for miR‐205.

## 1 INTRODUCTION

Cardiovascular diseases (CVDs) such as atherosclerosis are the leading cause of death globally.^1^, ^2^ The aberrant proliferation of vascular smooth muscle cells (VSMCs) plays the essential role in the pathogenesis of CVDs such as the development of atherosclerotic plaque.^3^, ^4^ Platelet‐derived growth factor (PDGF) exists as three isoforms including PDGF‐aa, PDGF‐ab and PDGF‐bb.^5^, ^6^ Studies have found that PDGF‐bb is overexpressed in the VSMCs in the atherosclerotic regions, and PDGF‐bb inhibition reduces the size of atherosclerotic lesion.^7^ In vitro mechanistic studies demonstrated that PDGF‐bb exerted enhanced effects on the VSMC proliferation and migration, and also promoted the extracellular matrix protein secretion of VSMCs,^8^ which may lead to the formation of atherosclerotic plaque. Up to date, the detailed mechanisms of PDGF‐bb‐mediated effects on the VSMCs remain elusive. Thus, finding novel mechanisms for PDGF‐bb is helpful for us to develop effective therapies for CVD.

Long non‐coding RNAs (lncRNAs) belong to the family of non‐coding RNAs, and lncRNAs have more than 200 nucleotides in length.^9^ Long non‐coding RNAs have been reported for the diverse functions in regulating cell proliferation, differentiation, metabolism, apoptosis and so on.^10^ Aberrant expression of various lncRNAs has been extensively reported in cancer studies, which emphasizes the importance of the lncRNAs in the cancer progression.^11^ Recently, it has also been reported that lncRNAs involve in the pathogenesis of CVDs. For example, the lncRNA metastasis‐associated lung adenocarcinoma transcript 1 (MALAT1) was decreased in human plaques and down‐regulation of MALAT1 was correlated with poor prognosis of CVDs.^12^ LincRNA‐p21 was reported to regulate VSMC proliferation and atherosclerotic formation via the enhancement of p53 activity.^13^ The lncRNA myosin heavy‐chain‐associated RNA transcripts were found to protect the heart from pathological hypertrophy.^14^ Up to date, the lncRNA small nucleolar RNA host gene 16 (SNHG16) has been well‐documented in several types of cancers including bladder cancer, liver cancer, cervical cancer, glioma and osteosarcoma.^15^, ^16^, ^17^, ^18^ However, the role of SNHG16 in CVDs has not been explored yet.

MicroRNAs (miRNAs) also belong to the family of non‐coding RNAs and have 21‐23 nucleotides in length, and miRNAs exerted transcriptional repression on the targeted genes via forming the imperfect binding with 3′ untranslated region (3′UTR) of the targeted genes.^19^ The role of miRNAs in the pathogenesis of CVDs has been reported in many studies.^20^ In terms of mechanistic actions of lncRNAs, lncRNAs can function as the competing endogenous RNAs (ceRNAs) for miRNAs, which in turn suppress the expression of targeted miRNAs. For example, lncRNA urothelial cancer associated 1 acts as ceRNA for miR‐26a to regulate VSMC proliferation and migration.^21^ LncRNA knockdown taurine‐up‐regulated gene 1 down‐regulates fibroblast growth factor 1 via acting as ceRNA for miR‐133a to attenuate atherosclerosis.^22^

In this study, we determined the expression of SNHG16 in human aortic smooth muscle cells (HASMCs) under the condition of PDGF‐bb and also explored the mechanistic role of SNHG16 in regulating HASMC proliferation and migration. More importantly, the clinical samples were collected from patients with atherosclerosis to analyse the expression of SNHG16 and its downstream targeting genes. Our data from this study may provide us with the advanced understanding of the molecular mechanisms of aberrant HASMC proliferation and migration.

## 2 MATERIALS AND METHODS

### 2.1 Oligonucleotides, cell transfections and chemicals

The SNHG16 overexpressing vector (pcDNA‐SNHG16) was synthesized by GenePharma Co. Ltd., and the pcDNA plasmid served as the negative control (NC). The siRNAs for SNHG16 and Smad2 as well as their corresponding scrambled siRNAs (si‐NC served as negative controls) were synthesized by RiboBio Co. Ltd. The miRNA oligonucleotides including miR‐205 mimic, miR‐205 inhibitor as well as their corresponding negative controls (mimic NC and inhibitor NC) were purchased from RiboBio Co. Ltd. Cell transfections with the oligonucleotides were performed with Lipofectamine 2000 reagent (Invitrogen) based on the manufacturer's protocol. The PDGF‐bb was purchased from AmyJet Scientific Co. Ltd., and the working concentration of PDGF‐bb for cell treatment was 20 ng/mL.

### 2.2 Cell line and cell culture conditions

The HASMCs were purchased from PromoCell, and the HASMCs were cultured in a smooth muscle cell growth medium (Sigma) and maintained in a humidified incubator supplied with 5% CO_2_ at 37°C.

### 2.3 Quantitative real‐time PCR (qPCR)

Total RNA from treated HASMCs and clinical plasma samples was extracted using TRIzol reagent (Invitrogen) based on the manufacturer's protocol. To quantify SNHG16 and Smad2 mRNA, the extracted RNA was reversely transcribed into cDNA using ThermoScript Reverse Transcriptase Kit (Invitrogen), and the real‐time PCR was performed with reverse transcription product on an Applied Biosystems 7900 Sequence Detection System (Applied Biosystems) using SYBR Green reaction kit (Takara). At the completion of the reactions, the Ct values were determined and the relative expression levels of SNHG16, proliferating cell nuclear antigen (PCNA) and Smad2 were calculated by the comparative Ct method using β‐actin as the internal control. To quantify miR‐205, the extracted RNA was reversely transcribed into cDNA using a stem‐loop RT primer (Applied Biosystems) and the AMV Reverse Transcriptase Kit (Takara, Dalian). The real‐time PCR was performed on an Applied Biosystems 7900 Sequence Detection System (Applied Biosystems) with a TaqMan PCR Kit (Takara). At the completion of the reactions, the Ct values were determined and the relative expression level of miR‐205 was calculated by the comparative Ct method using U6 as the internal control.

### 2.4 Cell Counting Kit‐8 (CCK‐8) assay

CCK‐8 assay was used to evaluate the cell viability of HASMCs after receiving different treatments. Briefly, the treated HASMCs were seeded into 96‐well plates at a density of 2 × 10^5^ cells/well, and the HASMCs were inducted with the CCK‐8 solution for 1 hour at room temperature. Then, the cell viability of HASMCs was determined by measuring the light absorbance at 450 nm on a microplate reader (Rayto Life and Analytical Science).

### 2.5 Transwell migration assay

Transwell migration assay was performed with Transwell inserts containing 8‐μm pore‐sized membrane (Merck Millipore). Briefly, the treated HASMCs were plated on upper chamber with serum‐free medium at a density of 5 × 10^6^ cells/well, while the lower chamber was filled with medium with 10% foetal bovine serum as the chemoattractant. The plated cells were allowed to migrate for 24 hour, and at 24 hour after incubation, the migrated cells were fixed with ice‐cold methanol followed by staining with crystal violet (0.1%). The number of migrated HASMCs was counted under a light microscope by randomly selecting five fields.

### 2.6 Wound healing assay

The wound healing assay was performed to determine the migratory potential of HASMCs. Briefly, treated HASMCs were plated on a 6‐well plate at a density of 5 × 10^5^ cells/well, and after 24 hour incubation, a straight‐line wound was created by scratching the bottom of well using a sterile 200‐µL pipette tip. The wound width at 0 and 24 hour after scratching was recorded, and the wound closure rate was calculated by (wound width at 0 hour – wound width at 24 hour)/wound width at 0 hour.

### 2.7 Dual‐luciferase reporter assay

The fragments of SNHG16 and Smad2 3′UTR were amplified by PCR using human genomic DNA, and the amplified fragments were subcloned into pGL3 reporter vector (Promega). The corresponding mutated fragments were generated using QuikChange II Site‐Directed Mutagenesis Kit (Agilent), and the mutated fragments were also inserted into the pGL3 reporter vector to construct the mutant reporter vector. For the luciferase reporter assays, HASMCs were seeded onto the 6‐well plates and cotransfected with firefly luciferase reporter plasmid, Renilla luciferase reporter plasmids (served as the normalizing control) and different miRNAs. Human aortic smooth muscle cells were collected at 48 hour after cotransfection and analysed for luciferase activity using a Dual‐Luciferase Reporter Kit (Promega).

### 2.8 RNA pull‐down assay

The RNA pull‐down assay was performed to determine the direct interaction between SNHG16 and miR‐205. Briefly, the commercially synthesized biotinylated SNHG16 probe (Bio‐SNHG16‐probe) or the negative control probes (Bio‐NC‐probe) were obtained from GenePharma and were incubated with Dynabeads M‐280 Streptavidin (Invitrogen) by following the manufacturer's instructions. After that, the beads that coated with different probes were further incubated with lysates of HASMCs. The interacted RNA complexes were then eluted and purified for further qRT‐PCR analysis.

### 2.9 Western blot assay

Proteins from treated HASMCs were extracted using RIPA buffer. Protein samples were detected by using antibodies against Smad2 (Cell Signaling Technology) and β‐actin (Cell Signaling Technology) according to previous studies for Western blot analysis.^23^ Protein blot bands were detected by ECL blotting substrate followed by exposing to an X‐ray film. The expression level of the Smad2 expression was determined by the densitometry method, and β‐actin was used as the internal control.

### 2.10 Clinical sample collection

The present study totally recruited 25 healthy volunteers and 25 patients with atherosclerosis. All the participants were from Sun Yat‐sen Memorial Hospital between 06/2015 and 12/2017. The plasma was collected from healthy volunteers who underwent routine physical examination in Sun Yat‐sen Memorial Hospital. The plasma was collected from patients with ischaemic stroke in the internal carotid artery underwent carotid vessel wall and brain magnetic resonance examinations within 1 week of symptom onset. All the patients had no cardioembolic stroke, haemorrhagic stroke, radiation therapy of the neck or other aetiologies such as vasculitis or moyamoya disease. All the studies were performed under the approval of the Ethics Committee of Sun Yat‐sen Memorial Hospital, and informed consent was obtained from all the participants.

### 2.11 Statistical analysis

All the data analysis was performed with the GraphPad Prism Software (version 6.0, GraphPad Software). All the experimental data were shown as mean ± standard deviation (SD). The statistical significance between different treatment groups was determined by Student's *t* test or one‐way ANOVA followed by Bonferroni's post hoc test. Spearman's correlation analysis was used for the determination of correlation between two parameters. The level of statistical significance was set at *P* < .05.

## 3 RESULTS

### 3.1 PDGF‐bb promoted cell proliferation and up‐regulated SNHG16 expression in HASMCs

**Figure 1:**
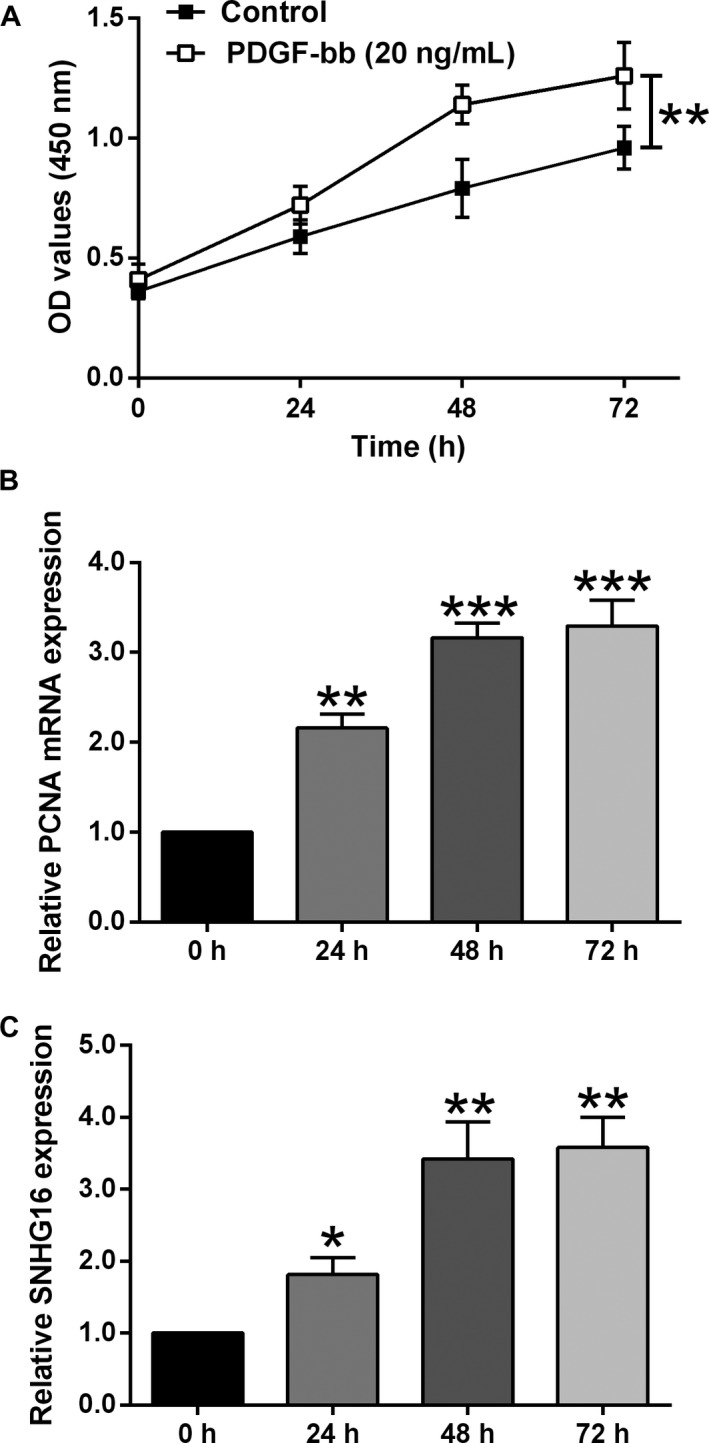
PDGF‐bb promoted cell proliferation and up‐regulated SNHG16 expression in HASMCs. A, The cell viability as measured by CCK‐8 assay was increased in HASMCs upon PDGF‐bb treatment. B, The expression level of PCNA mRNA as determined by qPCR was increased in HASMCs upon PDGF‐bb treatment. C, The expression level of SNHG16 as determined by qPCR was up‐regulated in HASMCs upon PDGF‐bb treatment. Data represent the mean ± SD (n = 3). Significant differences compared to control group were indicated as *P < .05, **P < .01 and ***P < .001

Firstly, we determined the cell viability of HASMCs after treated for different time durations, and PDGF‐bb significantly increased the cell viability of HASMCs compared to the control group (Figure 1A). Consistently, the mRNA expression level of PCNA was also significantly increased after being treated with PDGF‐bb for 24, 48 and 72 hour, respectively. Importantly, the SNHG16 expression was markedly up‐regulated in HASMCs received PDGF‐bb treatment for 24, 48 and 72 hour, respectively. As treatment with PDGF‐bb for 48 hour was effective in promoting HASMC proliferation and SNHG16 expression, treating HASMCs with PDGF‐bb for 48 hour was used in the following in vitro studies.

### 3.2 SNHG16 overexpression increased cell proliferation and migration of HASMCs

**Figure 2:**
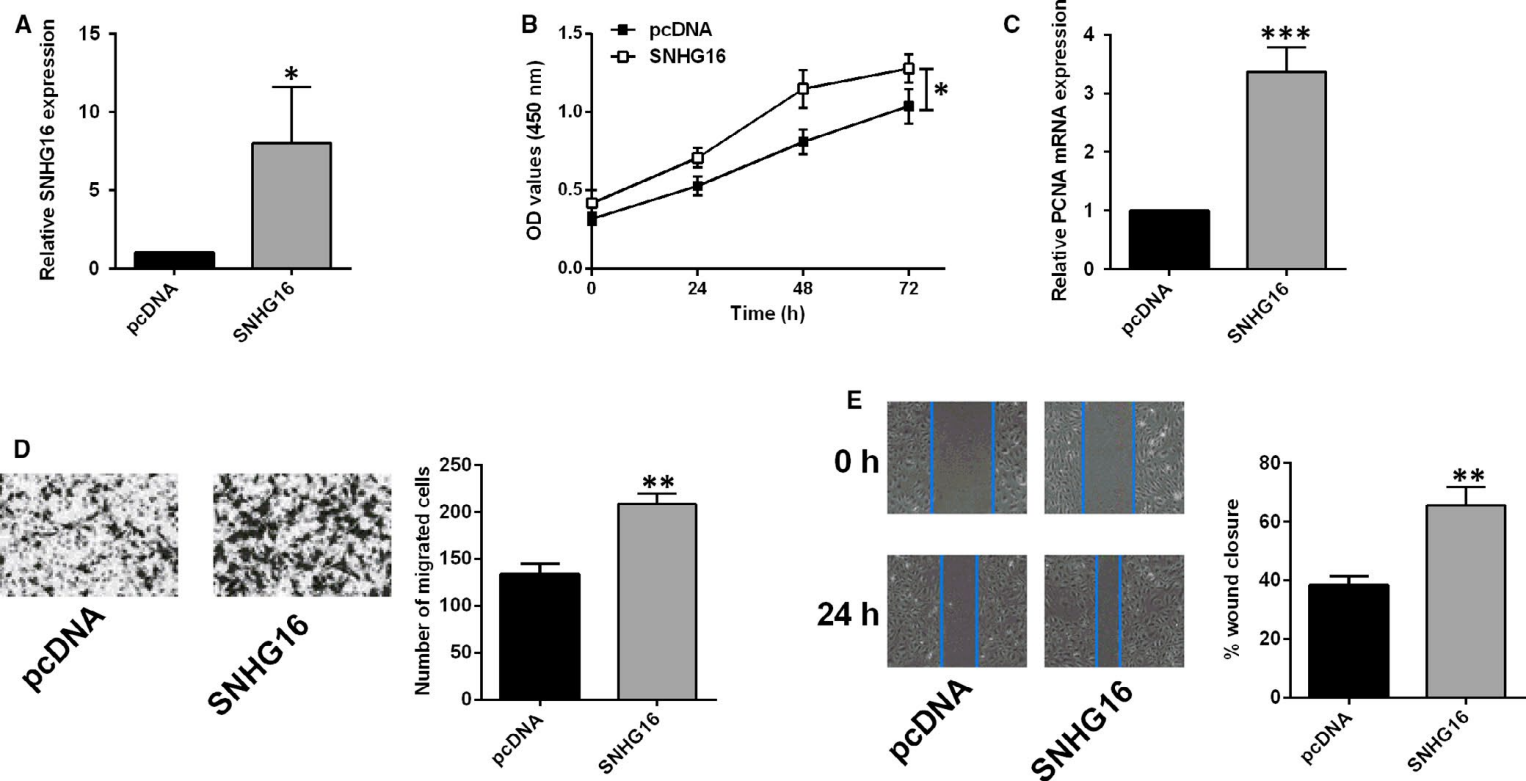
SNHG16 overexpression promoted cell proliferation and migration of HASMCs. A, The expression level of SNHG16 was increased in HASMCs upon pcDNA‐SNHG16 transfection. B, The cell viability as measured by CCK‐8 assay was increased in HASMCs upon pcDNA‐SNHG16 transfection. C, The expression level of mRNA as determined qPCR was increased in HASMCs upon pcDNA‐SNHG16 transfection. Cell migration as determined by Transwell migration assay (D) and wound healing assay (E) was increased in HASMCs upon pcDNA‐SNHG16 transfection. Data represent mean ± SD (n = 3). Significant differences compared to control group were indicated as *P < .05, **P < .01 and ***P < .001

The transient overexpression of SNHG16 was manipulated via transfecting HASMCs with pcDNA‐SNHG16, and qPCR assay showed that pcDNA‐SNHG16 transfection increased SNHG16 expression level by around eightfold when compared to pcDNA group (Figure 2A). The CCK‐8 assay showed that SNHG16 overexpression significantly increased the optical density values when compared to the control (pcDNA) group, suggesting that SNHG16 overexpression increased the cell viability of HASMCs (Figure 2B). Further qPCR assay showed that SNHG16 overexpression exerted enhanced effects on the PCNA mRNA expression (Figure 2C). Furthermore, the cell migration of HASMCs was assessed by two in vitro functional assays, that is Transwell migration and wound healing assays. As expected, SNHG16 overexpression significantly increased the number of migrated cells and promoted the wound healing (Figure 2D,E), suggesting SNHG16 exerted enhanced effects on the cell migratory potential of HASMCs.

### 3.3 SNHG16 knockdown suppressed cell proliferation and migration of PDGF‐bb‐treated HASMCs

**Figure 3:**
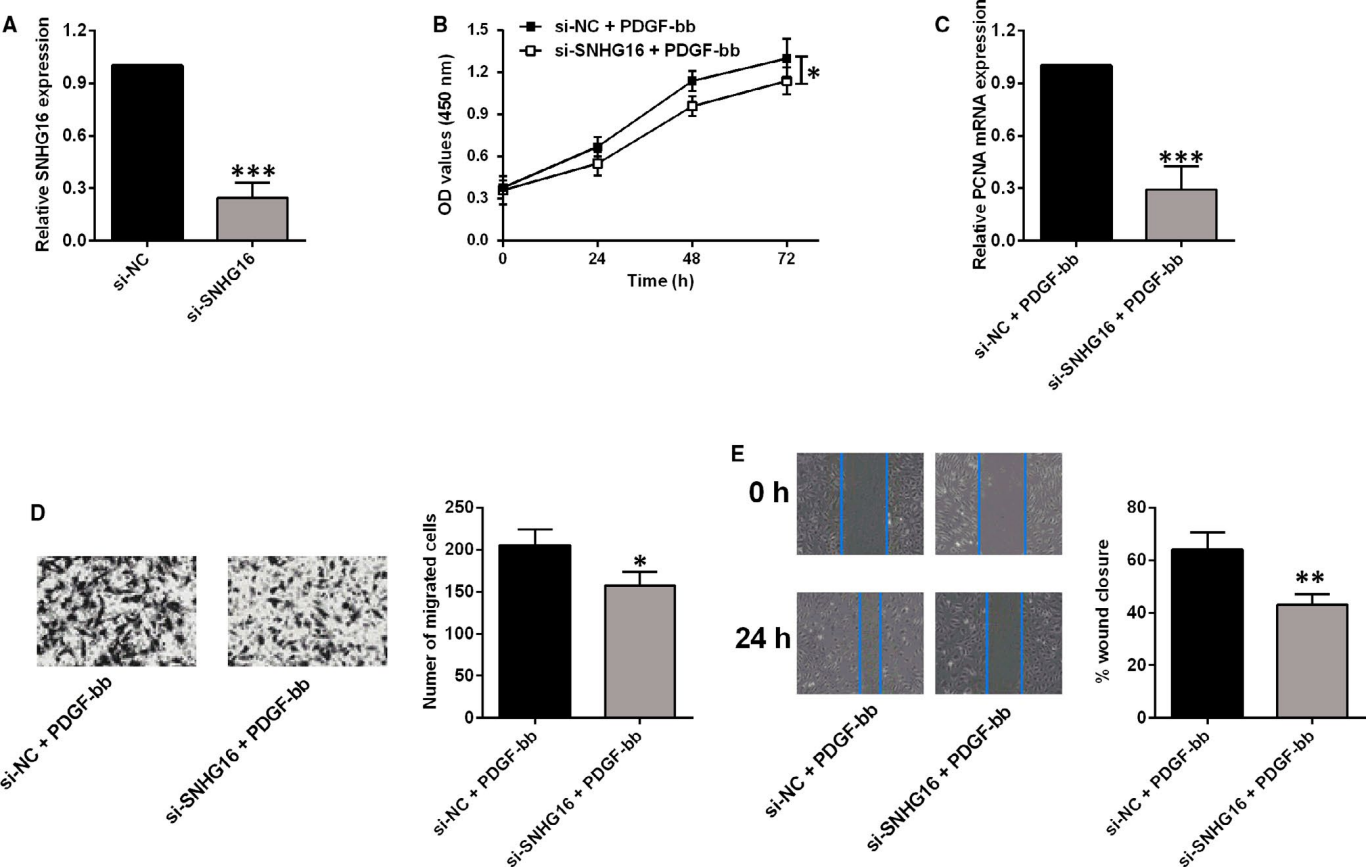
SNHG16 knockdown suppressed cell proliferation and migration of HASMCs treated with PDGF‐bb. A, The expression level of SNHG16 as measured by qPCR was suppressed in PDGF‐bb‐treated HASMCs upon SNHG16 siRNA transfection. B, The cell viability as determined by CCK‐8 assay was suppressed in PDGF‐bb‐treated HASMCs upon SNHG16 siRNA transfection. C, The expression level of PCNA mRNA as determined by qPCR was decreased in PDGF‐bb‐treated HASMCs upon SNHG16 siRNA transfection. Cell migration as determined by Transwell migration assay (D) and wound healing assay (E) was suppressed in PDGF‐bb‐treated HASMCs upon SNHG16 siRNA transfection. Data represent mean ± SD (n = 3). Significant differences compared to control group were indicated as *P < .05, **P < .01 and ***P < .001

The transient knockdown of SNHG16 was manipulated via transfecting HASMCs with SNHG16 siRNA, and qPCR assay showed that SNHG16 siRNA transfection down‐regulated SNHG16 expression when compared to cells transfected with scrambled siRNA (si‐NC) group (Figure 3A). In the PDGF‐bb‐treated HASMCs, SNHG16 knockdown significantly suppressed the cell viability as determined by CCK‐8 assay and down‐regulated PCNA expression as determined by qPCR (Figure 3B,C). In terms of HASMC migration, knockdown of SNHG16 reduced the number of migrated cells and attenuated the wound healing in the PDGF‐bb‐treated HASMCs (Figure 3D,E), suggesting the inhibitory effects of SNHG16 knockdown on cell migration in the PDGF‐bb‐treated HASMCs.

### 3.4 SNHG16 suppressed miR‐205 expression in HASMCs

**Figure 4:**
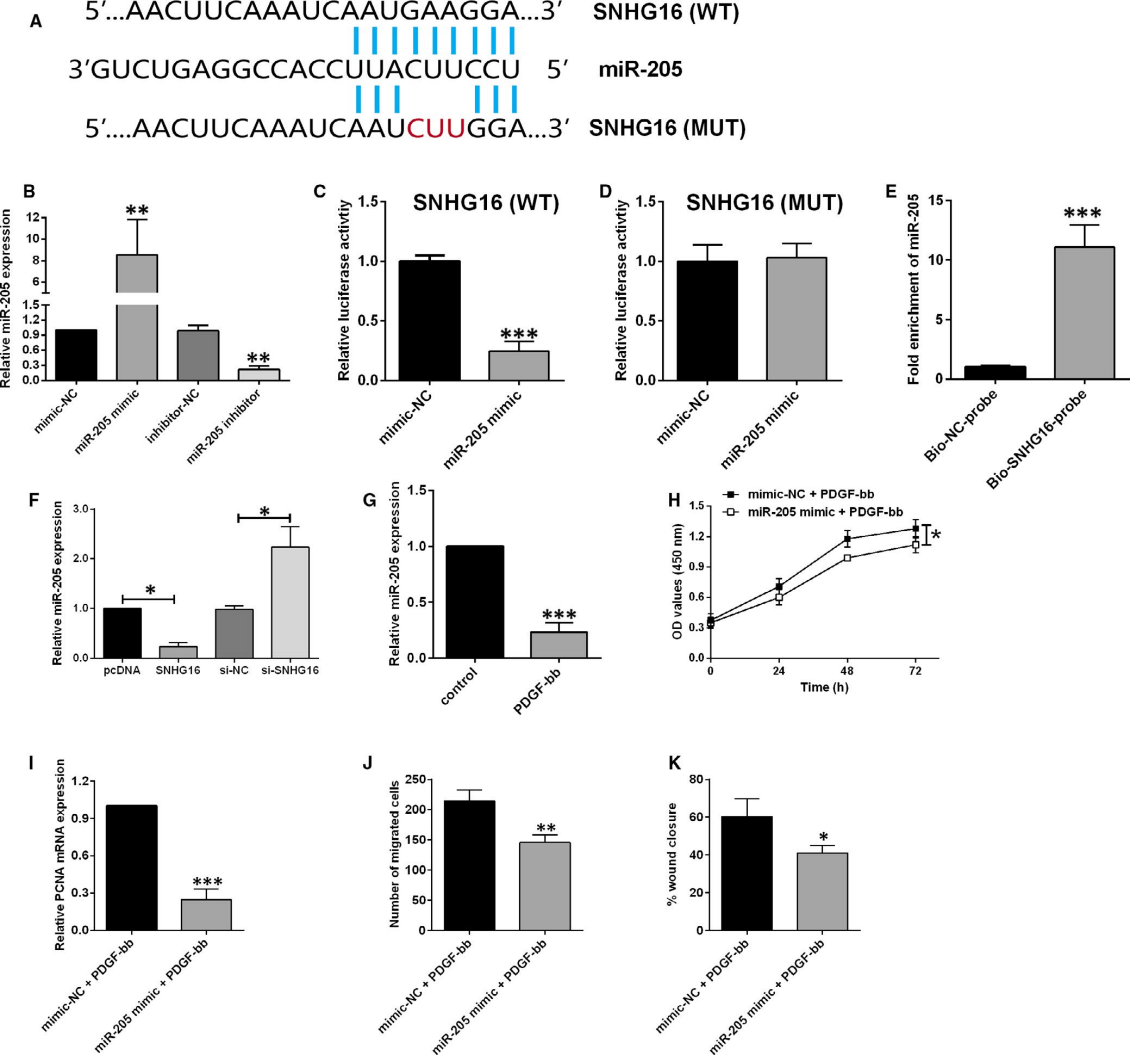
SNHG16 suppressed miR‐205 expression in HASMCs. A, The predicted binding regions between SNHG16 fragment and miR‐205. MUT, mutant; WT, wild type. B, The expression of miR‐205 as determined by qPCR was increased in HASMCs upon miR‐205 mimic transfection and was decreased in HASMCs upon miR‐205 inhibitor transfection. C, The luciferase activity of vector with wild‐type SNHG16 fragment was suppressed upon miR‐205 mimic transfection, while (D) the luciferase activity of the vector with mutant SNHG16 fragment was not affected. E, A significant amount of miR‐205 was detected in Bio‐SNHG16‐probe pulled down pellets in comparison with Bio‐NC‐probe group as determined by the RNA pull‐down assay. F, The expression level of miR‐205 was decreased by SNHG16 overexpression and was increased by SNHG16 knockdown, and (G) was suppressed by PDGF‐bb treatment in HASMCs. H, The cell viability as determined by CCK‐8 assay was inhibited by miR‐205 mimic transfection in PDGF‐bb‐treated HASMCs. I, The mRNA expression of PCNA as determined by qPCR was suppressed by miR‐205 mimic transfection in PDGF‐bb‐treated HASMCs. J and K, Cell migration as determined by Transwell migration assay and wound healing assay was suppressed in PDGF‐bb‐treated HASMCs. Data represent mean ± SD (n = 3). Significant differences compared to control group were indicated as *P < .05, **P < .01 and ***P < .001

To further explore the mechanistic role of SNHG16 in regulating HASMC proliferation and migration, we performed the bioinformatic analyses to determine the potential targets of SNHG16. By using the LncBase V2.0 Predicted, miR‐205 was identified as a potential target of SNHG16 (Figure 4A). To confirm the binding action between the predicted sites, we constructed the luciferase reporter vector either with wild‐type SNHG16 fragment or with mutant SNHG16 fragment (Figure 4A). The overexpression or knockdown of miR‐205 was manipulated by miR‐205 mimic or miR‐205 inhibitor transfection, respectively (Figure 4B). In terms of the luciferase activity, miR‐205 overexpression suppressed the luciferase activity of vector with wild‐type SNHG16 fragment (Figure 4C), but not with the mutant SNHG16 fragment (Figure 4D). The RNA pull‐down assay was further performed to confirm the direct interaction between SNHG16 and miR‐205, and a significant amount of miR‐205 was detected in Bio‐SNHG16‐probe pulled down pellets in comparison with Bio‐NC‐probe group (Figure 4E). The qPCR assay demonstrated that SNHG16 overexpression down‐regulated miR‐205 expression, while SNHG16 knockdown up‐regulated miR‐205 expression in HASMCs (Figure 4F). In addition, the expression level of miR‐205 was significantly suppressed in HASMCs after being treated with PDGF‐bb for 48 hour (Figure 4G). In terms of cell viability, miR‐205 overexpression inhibited the cell viability of PDGF‐bb‐treated HASMCs compared to mimic NC group (Figure 4H). The mRNA expression level of PCNA in PDGF‐bb‐treated HASMCs was also significantly suppressed by miR‐205 mimic transfection (Figure 4I). For the cell migration, miR‐205 overexpression significantly reduced the number of migrated cells and suppressed the wound healing in the PDGF‐bb‐treated HASMCs (Figure 4J,K).

### 3.5 MiR‐205 inversely regulated the expression of Smad2 in HASMCs

**Figure 5:**
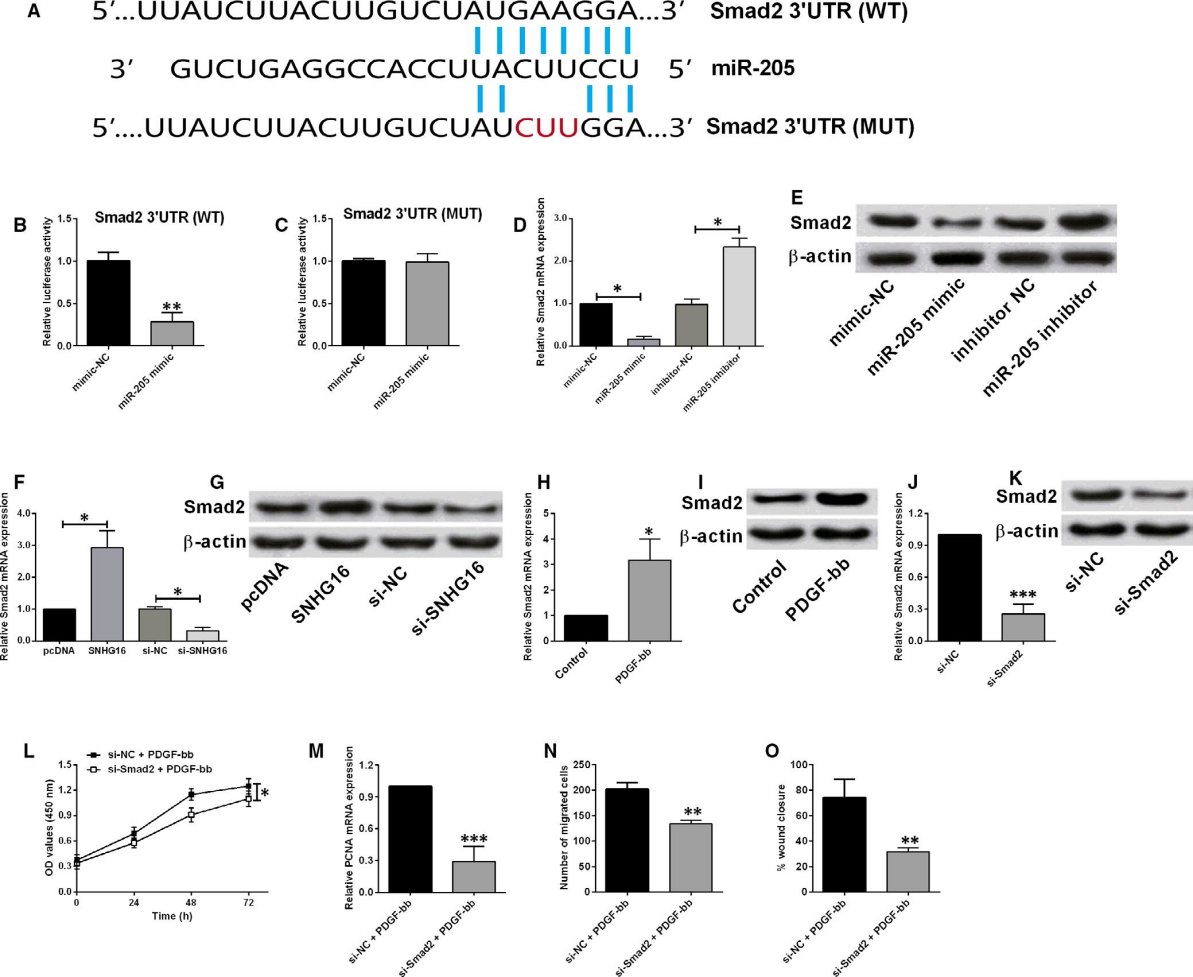
MiR‐205 inversely regulated Smad2 expression in HASMCs (A) the predicted binding regions between miR‐205 and Smad2 3′UTR. B, The luciferase activity of vector with wild‐type Smad2 3′UTR was decreased in HASMCs upon miR‐205 mimic transfection, while (C) the luciferase activity of vector with mutant Smad2 3′UTR was not affected. D, The mRNA and (E) protein expression levels of Smad2 were suppressed by miR‐205 overexpression and were increased by miR‐205 knockdown in HASMCs. F, The mRNA and (G) protein expression levels of Smad2 were increased by SNHG16 overexpression and were decreased by SNHG16 knockdown in HASMCs. H, The mRNA and (I) protein expression levels of Smad2 were increased in PDGF‐bb‐treated HASMCs. J, The mRNA and (K) protein expression levels of Smad2 were suppressed upon Smad2 siRNA transfection in HASMCs. L, The cell viability as determined by CCK‐8 assay was suppressed by Smad2 knockdown in PDGF‐bb‐treated HASMCs. M, The mRNA expression level of PCNA was suppressed by Smad2 knockdown in PDGF‐bb‐treated HASMCs. N and O, Cell migration as determined by Transwell migration and wound healing assays was suppressed by Smad2 knockdown in PDGF‐bb‐treated HASMCs. Data represent mean ± SD (n = 3). Significant differences compared to control group were indicated as *P < .05, **P < .01 and ***P < .001

The downstream targets of miR‐205 were further predicted by TargetScan software, and Smad2 was found to be a potential target of miR‐205. To verify the interaction between the predicted binding sites, we constructed the luciferase reporter vector with either wild‐type 3′UTR of Smad2 or mutant 3′UTR of Smad2 to determine the effects of miR‐205 on the luciferase activity (Figure 5A). Overexpression of miR‐205 significantly suppressed the luciferase activity of vector with wild‐type Smad2 3′UTR (Figure 5B), but not with the mutant Smad2 3′UTR in HASMCs (Figure 5C). Consistently, miR‐205 overexpression suppressed the mRNA and protein expression levels of Smad2, while miR‐205 knockdown increased the mRNA and protein expression levels of Smad2 in HASMCs (Figure 5D,E). Furthermore, SNHG16 overexpression up‐regulated Smad2 expression and SNHG16 knockdown down‐regulated Smad2 expression in HASMCs (Figure 5F,G). PDGF‐bb treatment for 48 hour also significantly increased the mRNA and protein expression levels of Smad2 in HASMCs (Figure 5H,I). The transient down‐regulation of Smad2 was manipulated via transfecting HASMCs with Smad2 siRNA, and Smad2 siRNA transfection markedly suppressed the mRNA and protein expression levels of Smad2 in HASMCs (Figure 5J,K). Further in vitro functional assays showed that knockdown of Smad2 suppressed cell viability, reduced PCNA mRNA expression and suppressed the cell migration in PDGF‐bb‐treated HASMCs (Figure 5L‐O).

### 3.6 SNHG16 regulated HASMC proliferation and migration via miR‐205/Smad2 axis

**Figure 6:**
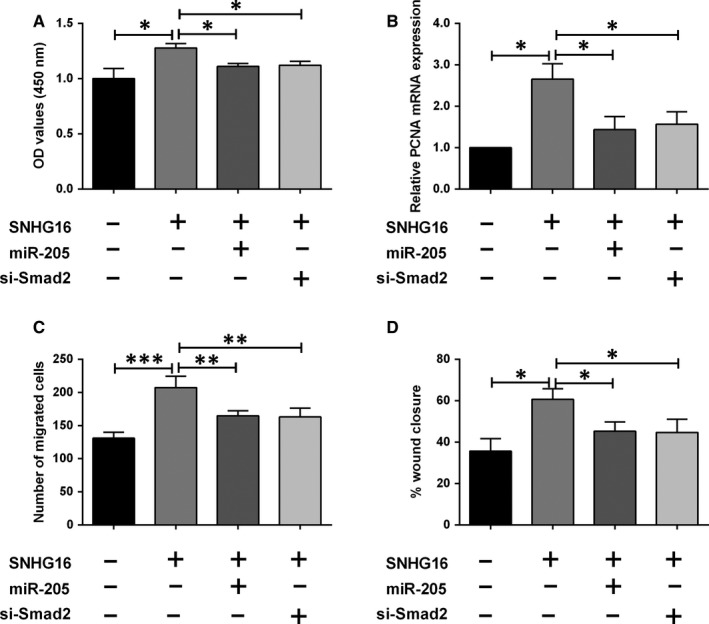
SNHG16 regulated HASMC proliferation and migration via miR‐205/Smad2 axis. A, The viability was determined by CCK‐8 assay in HASMCs after being treated with plasmid constructs, miRNAs or siRNAs. B, The mRNA expression of PCNA was determined by qPCR in HASMCs after being treated with plasmid constructs, miRNAs or siRNAs. C and D, Cell migration was determined by Transwell migration and wound healing assays in HASMCs after being treated with plasmid constructs, miRNAs or siRNAs. Data represent mean ± SD (n = 3). Significant differences compared to control group were indicated as *P < .05, **P < .01 and ***P < .001

As expected, SNHG16 overexpression alone increased cell viability and PCNA mRNA expression level and promoted cell migration in HASMCs when compared to negative control group (Figure 6A‐D). MiR‐205 overexpression by mimic transfection significantly attenuated the enhanced effects of SNHG16 overexpression on the HASMC proliferation and migration (Figure 6A‐D). Moreover, the knockdown of Smad2 also showed similar acting profiles, where the transfection of Smad2 siRNA significantly suppressed the cell proliferation and migration in HASMCs with SNHG16 overexpression (Figure 6A‐D).

### 3.7 The expression of SNHG16, miR‐205 and Smad2 in clinical samples from patients with atherosclerosis

**Figure 7:**
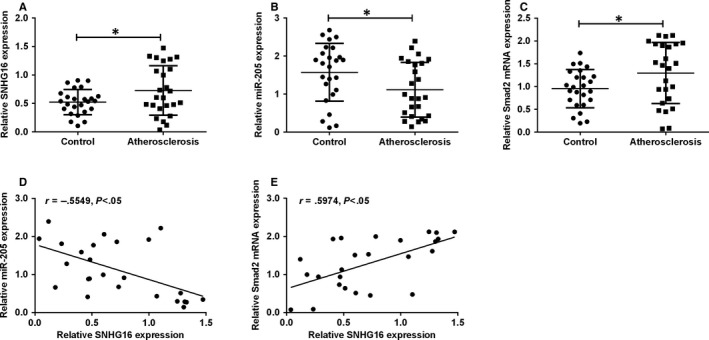
The expression of SNHG16, miR‐205 and Smad2 in clinical samples. A, The expression of SNHG16 as determined by qPCR was increased in plasma from patients with atherosclerosis. B, The expression of miR‐205 as determined by qPCR was decreased in the plasma from patients with atherosclerosis. C, The expression of Smad2 mRNA as determined by qPCR was increased in the plasma from patients with atherosclerosis. Data represent mean ± SD (n = 25). D, Correlation between SNHG16 expression and miR‐205 expression and (E) correlation between SNGH16 and Smad2 mRNA expression in the plasma from patients with atherosclerosis were analysed with Spearman's correlation analysis. Significant differences compared to control group were indicated as *P < .05

To further confirm the role of SNHG16 in the involvement of CVDs, we determined the expression levels of SNHG16, miR‐205 and Smad2 in the plasma samples from healthy volunteers and patients with atherosclerosis. The qPCR results showed that SNHG16 and Smad2 mRNA expression levels were up‐regulated in the plasma isolated from atherosclerotic patients when compared to healthy controls (Figure 7A,C), and the expression level of miR‐205 was significantly down‐regulated in the plasma from atherosclerotic patients (Figure 7B). In addition, SNHG16 expression was inversely correlated with miR‐205 expression (Figure 7D) and positively correlated with Smad2 expression in the plasma from atherosclerotic patients (Figure 7E).

## 4 DISCUSSION

The pathogenesis of atherosclerosis involves both genetic and environmental factors, and the abnormal cellular behaviours of VSMCs are believed to be the main contributor to the development of atherosclerosis.^4^ Therefore, identifying novel genes or signalling pathway for VSMC proliferation and migration is a reasonable strategy to provide better solutions for the treatment of atherosclerosis. In the present study, we found that PDGF‐bb treatment promoted cell proliferation and migration with concurrent up‐regulation of SNHG16 in HASMCs. Small nucleolar RNA host gene 16 overexpression promoted HASMC proliferation and migration, while SNHG16 knockdown suppressed cell proliferation and migration in the PDGF‐bb‐stimulated HASMCs. The bioinformatic analyses showed that SNHG16 possessed the complementary binding sequence with miR‐205, where the interaction was confirmed by luciferase reporter assay and RNA pull‐down assay in HASMCs, and SNHG6 inversely regulated the expression of miR‐205. MiR‐205 overexpression attenuated the enhanced effects of PDGF‐bb treatment on HASMC proliferation and migration. Moreover, Smad2 was identified as a target of miR‐205, and Smad2 was inversely regulated by miR‐205, but was positively regulated by SNHG16 in HASMCs. Smad2 knockdown also attenuated PDGF‐bb‐mediated actions on HASMC proliferation and migration. Further rescue experiments showed that both miR‐205 overexpression and Smad2 knockdown partially reversed the effects of SNHG16 overexpression on HASMC proliferation and migration. More importantly, the clinical findings were consistent with in vitro results, and SNHG16 and Smad2 mRNA were up‐regulated and miR‐205 was down‐regulated in the plasma from patients with atherosclerosis. In addition, SNHG16 expression was inversely correlated with miR‐205 expression and positively correlated with Smad2 expression in the plasma from atherosclerotic patients. Collectively, our results for the first time demonstrated the SNHG16/miR‐205/Smad2 axis in the regulation of HASMC proliferation and migration.

The lncRNA SNHG16 has been implicated for its important roles in regulating cancer progression, and various molecular mechanisms for SNHG16 were also proposed. The oncogenic role of SNHG16 was identified in several types of cancers including hepatocellular carcinoma,^16^ glioma,^24^ osteosarcoma,^18^ bladder cancer 25 and cervical cancer.^17^ Among these cancers, SNHG16 promoted the cancer cell proliferation and migration via distinct molecular mechanisms. The most common mechanistic actions of SNHG16 in regulating cancer progression are the ceRNA function to repress its downstream targeted miRNAs such as miR‐216, miR‐98, miR‐340 and miR‐302a‐3p. Consistently, our results also showed that SNHG16 was up‐regulated upon PDGF‐bb stimulation and SNHG16 overexpression promoted HASMC proliferation and migration, suggesting the enhanced effects of SNHG16 on the HASMC proliferation and migration.

As the SNHG16 was found to act as ceRNAs in the cancer studies, we further determined the ceRNA actions of SNHG16 in HASMCs, and miR‐205 was confirmed to be inversely regulated by miR‐205 in HASMCs. Previous studies have demonstrated that miR‐205 exerted tumour‐suppressive actions by inhibiting cancer cell proliferation and metastasis in various types of cancers.^26^ In the cardiovascular research, miR‐205 suppressed cell proliferation, migration and tube formation of endothelial cells,^27^ and miR‐205 also exerted suppressive effects on the osteoblastic differentiation and calcification of VSMCs.^28^ In agreement with previous findings, our data showed that miR‐205 overexpression suppressed proliferation and migration of HASMCs with PDGF‐bb treatment, and the enhanced effects of SNHG16 overexpression on HASMC proliferation and migration were attenuated by miR‐205 overexpression, implying that SNHG16 exerted its positive actions on HASMC proliferation and migration at least via targeting miR‐205.

As miRNA is well‐known for its mechanism in which miRNA targets 3′UTR of the targeted gene and subsequently represses the gene expression. In this regard, we performed bioinformatic analyses and found many potential targets for miR‐205. Among these targets, we selected Smad2 for further investigation as Smad2 has been reported for its role in regulating cellular functions of VSMCs.^29^ Mao et al found that knockdown of Smad2 reduced cell proliferation, migration and attachment of VSMCs.^30^ Samd2 also increased the expression level of differentiation marker genes in VSMCs.^31^ Further studies showed that Smad2‐mediated VSMC proliferation may involve the TGFβ and AKT signalling pathways.^32^ Consistently, Smad2 knockdown suppressed proliferation and migration in PDGF‐bb‐stimulated HASMCs, and also attenuated the hyper‐proliferative and migratory ability of HASMCs with SNHG16 overexpression. Importantly, the investigation of clinical plasma samples from patients with atherosclerosis showed the up‐regulation of SNHG16 and Smad2, and down‐regulation of miR‐205. All these results lead us to suggest that SNHG16 promoted HASMC proliferation and migration via partly acting on the miR‐205/Smad2 axis.

The present study has some limitations. First of all, the study focused on the in vitro mechanistic studies, and there is a lack of animal studies. Thus, in vivo studies will be our future directions to look at the therapeutic effects of targeting SNHG16 in an animal of atherosclerosis. Secondly, the clinical sample size was relatively small, and increased sample size should be considered to confirm our current findings. Thirdly, PDGF‐bb was used to induce the up‐regulation of SNHG16; however, other pathogenic stimuli may also involve the development of atherosclerosis. Thus, other stimuli may be also investigated to provide a full understanding of the molecular mechanisms of SNHG16 in regulating HASMC proliferation and migration.

In conclusion, our data showed the up‐regulation of SNHG16 in pathogenic‐stimulated HASMCs and clinical samples from atherosclerotic patients. Further detailed investigation using different in vitro functional assays revealed the enhance effects of SNHG16 on HASMC proliferation and migration, and SNHG16 exerts its effects via regulating Smad2 overexpression by acting as a ceRNA for miR‐205. Whether targeting SNHG16 could provide effective therapy for atherosclerosis may require further investigation.

## CONFLICT OF INTEREST

The authors declare that there is no conflict of interest.

## AUTHOR CONTRIBUTION

YQL and YL designed the experiments; YQL, GT, HZ and WY performed the experiments and analysed the data; YX, YY and JW were involved in the interpretation of the results. YQL and YL wrote the paper. All authors read and approved the final manuscript.

## Data Availability

The data sets generated and/or analysed during the current study are available from the corresponding author on reasonable request.
